# Circulating tumor DNA as an emerging liquid biopsy biomarker for early diagnosis and therapeutic monitoring in hepatocellular carcinoma

**DOI:** 10.7150/ijbs.44024

**Published:** 2020-03-05

**Authors:** Xiaolin Wu, Jiahui Li, Asmae Gassa, Denise Buchner, Hakan Alakus, Qiongzhu Dong, Ning Ren, Ming Liu, Margarete Odenthal, Dirk Stippel, Christiane Bruns, Yue Zhao, Roger Wahba

**Affiliations:** 1 Department of General, Visceral, Cancer and Transplantation Surgery, University Hospital of Cologne, Kerpener Straße 62, 50937, Cologne, Germany.; 2 Department of Cardiothoracic Surgery, Heart Center, University Hospital of Cologne, Germany, Kerpener Straße 62, 5.937 Cologne, Germany.; 3 Department of General Surgery, Huashan Hospital & Cancer Metastasis Institute & Institutes of Biomedical Sciences, Fudan University, 200032, Shanghai, P.R. China.; 4 Liver Cancer Institute & Zhongshan Hospital; Department of Surgery, Institute of Fudan-Minhang Academic Health System, Minhang Branch, Zhongshan Hospital, Fudan University, 200032, Shanghai, P.R. China.; 5 Affiliated Cancer Hospital & Institute of Guangzhou Medical University; Key Laboratory of Protein Modification and Degradation, School of Basic Medical Sciences, 510095, Guangzhou, P.R. China.; 6 Institute of Pathology, University Hospital of Cologne, 50937, Cologne, Germany.; 7 Department of General, Visceral und Vascular Surgery, Otto-von-Guericke University, 39120, Magdeburg, Germany.

**Keywords:** circulating tumor DNA, cell-free DNA, liquid biopsy, biomarker, hepatocellular carcinoma, liver cancer

## Abstract

As one of the most common malignant tumors worldwide, hepatocellular carcinoma (HCC) is known for its poor prognosis due to diagnosis only in advanced stages. Nearly 50% of the patients with the first diagnosis of HCC die within a year. Currently, the advancements in the integration of omics information have begun to transform the clinical management of cancer patients. Molecular profiling for HCC patients is in general obtained from resected tumor materials or biopsies. However, the resected tumor tissue is limited and can only be obtained through surgery, so that dynamic monitoring of patients cannot be performed. Compared to invasive procedures, circulating tumor DNA (ctDNA) has been proposed as an alternative source to perform molecular profiling of tumor DNA in cancer patients. The detection of abnormal forms of circulating cell-free DNA (cfDNA) that originate from cancer cells (ctDNA) provides a novel tool for cancer detection and disease monitoring. This may also be an opportunity to optimize the early diagnosis of HCC. In this review, we summarized the updated methods, materials, storage of sampling, detection techniques for ctDNA and the comparison of the applications among different biomarkers in HCC patients. In particular, we analyzed ctDNA studies dealing with copy number variations, gene integrity, mutations (RAS, TERT, CTNNB1, TP53 and so on), DNA methylation alterations (DBX2, THY1, TGR5 and so on) for the potential utility of ctDNA in the diagnosis and management of HCC. The biological functions and correlated signaling pathways of ctDNA associated genes (including MAPK/RAS pathway, p53 signaling pathway and Wnt-β catenin pathway) are also discussed and highlighted. Thus, exploration of ctDNA/cfDNA as potential biomarkers may provide a great opportunity in future liquid biopsy applications for HCC.

## Introduction

Hepatocellular carcinoma (HCC) is one of the most lethal cancers worldwide with progressive accumulation and poor prognosis. Early diagnosis is crucial in HCC because it provides multiple curative therapeutic options: liver transplantation, liver resection or microwave ablation, in addition, survival could also be prolonged by transarterial chemoembolization (TACE), systemic therapy with tyrosine kinase inhibitor and selective internal radiation therapy (SIRT) 1. Patients with different stages of HCC show huge differences in prognosis. At early stage(I) patients with HCC compared to patients in advanced stage (III) shows a significant improved 5-year survival rate with 59% compared to 29% 2. Unfortunately, HCC is asymptomatic at an early stage, and the majority of HCC is detected in the palliative stage. Therefore, the early diagnosis of HCC can only rely on modern medical technology. At present, the clinical practice includes radiological screening and monitoring for patients with defined risk factors (liver cirrhosis, viral or chronic hepatitis, NAFLD, etc.) in combination with AFP measurement. AFP is one of the most widely used tumor markers for HCC. With a low sensitivity of 62.4% and a cut-off value of 20 ng/ml, AFP is not sensitive and accurate enough for early detection and may reveal false-negative results 3,4. Imaging techniques including (CT, MRI or CEUS) had improved the sensitivity from 66% to 82% and the specificity to more than 90% merely for detecting nodules with at least 1cm diameter 5.

Liquid biopsy could be a future alternative strategy. In cancer research, it has developed rapidly as a diagnostic and monitoring tool, which can be easily collected and analyzed in non-solid biological tissue. The term “liquid biopsy” encompasses circulating tumor DNA (ctDNA)/cell-free DNA (cfDNA), circulating tumor cells (CTCs), circulating miRNAs, and exosomes 6,7. In this context, ctDNA/cfDNA is one of the most frequently analyzed objects.

First reported in human peripheral blood in 1948 by Mandel and Metais 8, cfDNA is found as double-stranded fragments of approximately 150 to 200 base pairs in length 9, with a half-life of less than an hour. CfDNA, from myeloid and lymphoid apoptotic cells, shows low levels in healthy individuals (averagely 10 to 15 ng per milliliter 10). The concentration of cfDNA can rise in the blood in cases of carcinoma, surgery, inflammation and tissue damage.

Circulating tumor DNA (ctDNA), which merely refers to fragmented DNA, originates from tumor cells itself. It represents a part of cfDNA, although ctDNA has a substantial fluctuant proportion ranging from <0.1% to >90% in cfDNA 11, it is more specific. In general, cfDNA levels in the blood are elevated in patients with carcinoma compared to healthy individuals. With lots of ctDNA released into the circulatory system by tumor cell apoptosis or necrosis, the quantity of ctDNA could reflect tumor burden in cancer patients. How to detect and analyze ctDNA from the background of normal cfDNA is a big challenge in the development of liquid biopsy applications and should be discussed in this review.

## Methods and materials

A systematical search in PubMed for the terms “liquid biopsy”, “cell-free DNA”, “circulating tumor DNA” in combination with “liver cancer”, “hepatocellular carcinoma”, “biomarkers”, including all papers in English from 2013-2019 has been performed. To summarize the latest developments, we elaborate on an overview of ctDNA in HCC with aspects about storage, detection, clinical application, biomarkers, gene alterations and the function of the alternated genes.

### Storage and detection methods for cfDNA

The most common source for the extraction of cfDNA is peripheral blood. CfDNA could also be detected in other body fluids, including saliva, ascites, pleural effusion, urine or stool. Currently, it is still widely discussed, if cfDNA should be extracted from serum or plasma. Some studies discovered that the level of cfDNA was higher in serum compared to plasma 12; because a part of cfDNA is released during blood cell lysis in the clotting process in tubes before centrifugation, which leads to contamination 13. Thus, plasma is the preferable biological sample in most studies.

Due to the short half-life of cfDNA, specialized approaches are required for storage and extraction. Plasma should be separated no more than 4-6 hours by centrifugation in EDTA tubes, which are the recommended blood collection tubes 14. The plasma sample can be isolated from the whole blood by performing a 2000 x g centrifugation. However, when the blood has to be stored more than 6h, specialized cfDNA collection tubes (BCT and CellSave tubes) with fixatives are better. They can help to keep the cfDNA quality up to 96h, including high copy numbers 15. Temperature is also an essential factor affecting the quality of cfDNA. Recently, a study for cfDNA preservation found out that the background of cfDNA in the blood of the EDTA tube has a rise delay at 4°C 16. Storage at low temperature could improve the quality of blood specimens: the recommended storage conditions for samples are feezing at -80°C.

Techniques for the detection of cfDNA require high sensitivity and specificity due to the low cfDNA concentration and the lower level of ctDNA in the blood. Then cfDNA can be detected to identify several common DNA based changes, including copy number variations (CNV), DNA mutation, gene methylation, and gene fusions, which is reflecting the status of DNA in the tumor cells. Different kits extract cfDNA base on different methods, including silica-based columns and magnetic beads. The principles of these methods are based on the characteristics of cfDNA: the binding affinity of cfDNA molecules could be enriched by silica column and magnetic enrichment is for the negatively charged phosphate backbone of cfDNA 17,18. Right now the evidence in which extraction methods are optimal is rare. The evaluation of extraction efficiency and quality depends on the kits. Kit extraction may exhibit different extraction efficacy even using the same method 17.

The detection methods for cfDNA with different sensitivity and nucleotide coverage can be summarized as BEAMing [beads, emulsion, amplification and magnetics] 19, droplet digital PCR and sequencing methods (Sanger sequencing, next-generation sequencing [NGS]) 20. The changing rules of the sensitivity and nucleotide coverage in these analyses technology are that the less sensitivity, the more extended nucleotide coverage, and vice versa 11, which can be observed clearly in dd PCR and NGS techniques.

Digital droplet PCR (dd PCR), the third generation of the PCR technique, is a high-throughput technique. It provides high precision and sensitivity (0.01% or less for allele frequencies in cfDNA) 21. On the other hand, due to the high sensitivity, ddPCR is unable to cover a large number of sequences. NGS can form a gene panel with many sequences from whole-exome sequencing to targeted sequencing of cfDNA, which can optimize the limits of detection. In the research on the same batch of samples with two detection techniques, ddPCR and NGS have shown different characteristics. With the ascendant sensitivity, ddPCR revealed a higher detection rate than NGS (38% and 14%) in somatic mutations of cfDNA. It also took a precondition that objective mutation sites must be certainly known in advance. In other words, NGS is more suitable for primary gene profiling on multi-gene panels 22.

However, the sensitivity and specificity of the NGS methods could be constrained by the error rate of DNA polymerase and sequencing reactions. Molecular barcoding methods can help to tag the original template cfDNA with unique nucleotide barcode and allow correct gene changes to be accurately identified. In the original research, it is necessary to make a rational choice for different methods according to different objectives 23.

### Different biomarkers in ctDNA of HCC

Due to the extraordinary amount and variability of ctDNA in the cfDNA background, it is important to detect and distinguish effective ctDNA from normal cfDNA accurately. This turns to be the challenge of promoting this technology in clinical diagnosis and treatment. As the fragmented DNA is released from carcinoma cells, ctDNA carries tumor-specific genetic information or epigenetic changes, which cannot be found in normal cfDNA. This helps to distinguish ctDNA from cfDNA.

The molecular changes that are useful to be biomarkers of ctDNA must fulfill the following criteria:

- The biomarkers should be detected in tumor cells and plasma DNA simultaneously in the same individual;
- The biomarkers must not be detected in cfDNA of healthy individuals.

Significant genetic and epigenetic information alterations in tumors include ctDNA level, CNVs, gene integrity, gene mutations, DNA methylation alterations and gene fusions, which can be divided into quantitative determination and qualitative alteration. Commonly altered genes may be different even in the same patient and also cohort depending on the type of genetic alteration. For example, most commonly mutant genes are TP53 and CTNNB1, while the most common amplifications are found in MET and CCND1 24.

Currently, many studies in the last five years have investigated the specific gene changes from HCC tumor tissues that can also be observed in ctDNA as potential specific biomarkers: ctDNA level, CNVs, gene integrity, gene mutations and DNA methylation alterations (Table 1). The majority of the data are gathered from Asian cohorts.

#### CfDNA quantitative determination

CfDNA quantitative determination focuses on cfDNA level and ctDNA copy numbers variation. The increase of cfDNA level and copy number in tumor patients are explained by the raised number of apoptotic and necrotic carcinoma cells. It has been proven in liver cancer, oesophageal cancer, breast cancer, and pancreatic cancer 22,52,53. Recently, Yan L et al. clearly showed that the cfDNA concentration in plasma was significantly higher in HCC patients compared to patients with liver fibrosis or healthy individuals 25. And Xu H et al. also discovered a more significant CNV score in HCC patient group than the chronic liver disease group 26.

#### Gene integrity

Gene integrity analysis is also an efficient way to distinguish ctDNA from the non-tumoral cfDNA in the plasma of HCC patients, focusing on the size of ctDNA molecules. Tumor-derived DNA molecules in plasma have a particular size profile. Jiang P et al. in 2015 illustrated that the size of ctDNA molecules in peripheral blood of HCC patients was smaller than those from healthy individuals. Short DNA molecules may preferentially carry the tumor-associated gene aberrations 29. Furthermore, ctDNA did not fragment randomly, because a class of ctDNA signatures forming preferred plasma DNA end coordinates. Thus ctDNA end coordinates could be served as hallmarks of DNA fragments from tumors as well 31.

#### Gene mutation

The number of somatic mutation sites in ctDNA reflects tumor burden in most HCC patients and represents the gene information of the primary cancer biopsy as well. In European HCC patient cohorts with large carcinoma (>5cm) or metastasis, at least one mutation could be detected in 86% of the cases showing that the genetic information in ctDNA reflected the condition of the primary tumor 34. Lim HY et al. showed further details of gene mutation in HCC patients: 4.4% mutation for RAS could be detected by beaming technology. TERT, TP53, and CTNNB1 mutation could be detected in peripheral blood of HCC patients with RAS mutations confirmed by Beaming technology before. The frequencies of detected mutations of ctDNA corresponded with the somatic mutations in the hepatocellular carcinoma were 44% (RAS), 63.0% (TERT), 48.1% (TP53), and 37.0% (CTNNB1) respectively 32. In Chinese cohorts with high HBV infection frequency, TP53 had the highest mutation rate (60.0%). Frequently mutated genes were CTNNB1 (15.7%), AXIN1 (14.3%), and ARID1A (14.3%), while TERT mutation was not included in the target deep sequencing (TDS) in the original design. In total, 38.6% of HCC patients carried a mutation gene, which could be a potential direction for discovering novel therapeutic targets 35. Simultaneously, the frequency of common gene mutations in HCC may vary in different cohorts. For example, ARID1A was detected as the gene with the highest mutation rate in ctDNA of HCC patients in some European cohorts 36 instead of TP53.

General mutation genes (RAS, TERT, CTNNB1, TP53, AXIN, ARID1A) detected in ctDNA with a high incidence rate, were also in the top genomic panel which was determined to be significantly mutated genes (SMGs). In HCC nodules, the oncogenes-TERT, CTNNB1, KRAS and NRAS mutations were present in 39%, 27%, 1% and 1% respectively 54, 55. Tumor suppressor genes TP53 (31%) and AXIN1 (8%) were inactivated by mutation, as well as the chromatin remodeling genes ARID1A (7%) 55.

**Figure 1:**
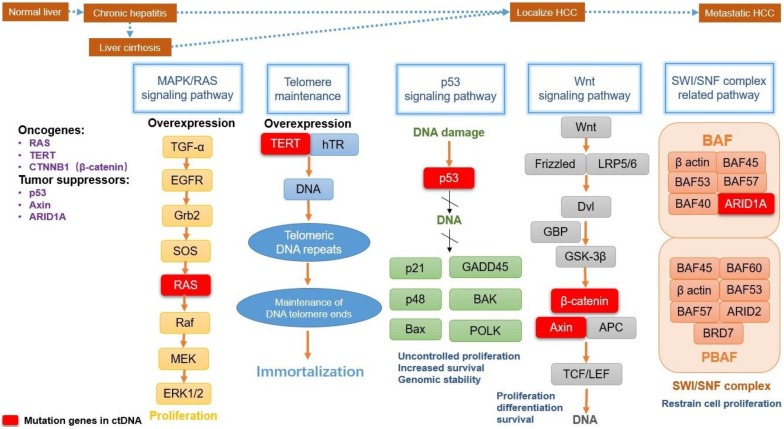
Main mutation pathways and functions in HCC development. MAPK/RAS pathway(marked with yellow), TERT mutation(marked with blue), p53 signaling pathway(marked with green), Wnt-β catenin pathway(marked with gray), and SWI/SNF complex related pathway(marked with light red) are the common centralized signaling pathways. They affect tumorigenesis and progression of hepatocellular carcinoma, involving several common oncogenes and tumor suppressors. Corresponding functions include proliferation, immortalization, genomic stability, cell differentiation and survival. The mutation genes detected in ctDNA show significant roles in pathways (Red box).

These mutant genes in ctDNA are involved in several major signaling pathways. They play a key role in the tumorigenesis and progression of HCC (summarized in Fig. 1), including MAPK/RAS pathway, Telomere maintenance, p53 signaling pathway, Wnt-β catenin pathway, and SWI/SNF complex related pathway 56.

The MAPK/RAS is a ubiquitous signaling pathway in all eukaryotic cells, which contains a chain of proteins. It is located downstream of many growth factors for signal transduction from the cell surface to the DNA in the cell nucleus. Therefore, MAPK/RAS pathway plays a fundamental role in the processes of cell proliferation and survival for many malignant tumors, including HCC, non-small cell lung cancer, renal cell carcinoma or melanoma 57. RAS gene belongs to oncogenes including KRAS, NRAS, and HRAS, separately coding KRAS, NRAS, and HRAS proteins in the RAS family 58. As a small molecular-weight GTP-binding protein, the RAS family is crucial for cell growth control. RAS is an important molecular signal regulator within this pathway. Once RAS is active, it interacts with some effectors to influence Raf protein kinases. Then the mitogen-activated protein (MAP) kinase cascade is activated by Raf, causing ERK activation to control the cell cycle. At the ends of human chromosomes, telomerase is the cap for protection and could lengthen cell longevity. Telomerase reverse transcriptase (TERT; or hTERT) is a catalytic subunit of the telomerase complex and has dominant roles for telomerase activity. Telomerase is inactivated in most somatic cells and it will be reactivated only in proliferative cells and most cancer cells, including liver cancer cells. The TERT expression in cancer cell survival can be upregulated in HCC, especially in patients with hepatitis C virus infection 54. Currently, TERT can be a gatekeeper in the whole process from chronic hepatitis to HCC. In liver cirrhotic tissues, TERT promoter mutation has been able to identify in dysplastic nodules 59. Tumor protein p53 (TP53), also called p53, is classified as a tumor suppressor gene. In humans, TP53 is an adapter in the DNA repair protein and can facilitate damaged DNA repair by checkpoint arrest in the cell cycle. Mutations in the TP53 signaling pathway are widespread in all kinds of tumors, including HCC. Common viruses and chemicals etiologic factors of HCC can prime TP53 mutations, including infection with HBV and HCV, exposure particularly aflatoxin B and chronic inflammation 60. TP53 mutation causes DNA damage repair to be hindered, leading tumor suppression is compromised and “gain function” in HCC cells, including uncontrolled proliferation, drug resistance, and cancer cell migration. TP53 mutation of ctDNA has a high detection rate in HCC notwithstanding that, the evidence for TP53 mutation in HCC ctDNA as tissue-specific is rare, because of the prevalence in multiple malignant tumors. The Wnt signaling pathway is an important cascade that contains a series of signal transducer factors for signal delivery. With the effect of stemness and development regulation, Wnt is one of the most frequently activated pathways associated with HCC progression 61. The oncogene β-catenin (CTNNB1) and the tumor suppressor Axin play a crucial role in the Wnt signaling pathway. In canonical Wnt signaling with gene transcription regulation, transcriptional co-activator CTNNB1 will help to stimulate gene expression, causing cell proliferation, anti-apoptosis and angiogenesis 62. Furthermore, CTNNB1 can cooperate with TERT to induce liver cell transformation. The tumor suppressors gene Axin is a scaffolding protein, which has been proved to be a negative regulator of the Wnt pathway a long time ago. However, there has been a study showed Axin mutation may be independent of Wnt/β-catenin pathway in some HCC paitents 63. It is not accurate enough to classify liver tumors with activating CTNNB1 mutation and inactivating Axin mutation into the “Wnt/β-catenin” group at the same time. ARID1A is a key subunit in SWI/SNF complex (Switch/Sucrose Non-Fermentable) for chromatin-remodeling, and the ARID1A gene also mutated frequently in liver carcinoma patients. SWI/SNF complex contains two large subclasses: BAF and PBAF. The BAF subunit ARID1A may take the highest mutation rate among other SWI/SNF components. As a tumor suppressor gene, ARID1A has been confirmed to restrain cell proliferation. On the other US-Data show that the expression levels of ARID1A are negatively correlated with survival level in patients with HCC. This may indicate a dual role of the ARID1A gene in tumorigenicity and cancer suppression for different temporal and cellular background in HCC 64.

#### Gene methylation alterations

Mammalian DNA methylation alterations at the 5-position of cytosine (5 mC), a vital epigenetic regulator of gene expression profile occur in CpG sites (cytosine-phosphate-guanine sites). It usually leads to alternations in DNA conformation, chromatin structure, and DNA protein interactions. DNA methylation causes gene silencing and genome imprinting. As DNA epigenetic modification that has been linked to DNA regulation and carcinoma pathogenesis, it could appear in the early stage of tumor development. Therefore, DNA methylation is a biomarker in various human cancer types. Currently, the elevation of methylation hallmarks from ctDNA in the plasma is one of the most extensively investigated fields in liquid biopsy in HCC. In the last 5 years, nearly 50% of the studies about biomarkers in HCC focus on gene methylation. Studies on methylation biomarkers in ctDNA can be divided into three categories: methylation sites quantity, methylation site expression and 5-hydroxymethylcytosine (5 hmC) detection.

A method, called 'CancerDetector' was used to probabilistically model the joint methylation states of multiple adjacent CpG sites on an individual sequencing read, in order to exploit the pervasive nature of DNA methylation generated for signal amplification. This could detect methylation sites sensitively and further identify cancer at an early stage 42.

Besides the amount of multiple CpG and 5hmC, a large number of independent hypermethylated gene sites in ctDNA from HCC patients were identified, such as DBX2, THY1, TGR5, MT1M, MT1G, INK4A, VIM, FBLN1, RGS10, ST8SIA6, RUNX, and SEPT9. DBX2 and THY1 methylation sites were proved to be detectable in ctDNA by Infinium Human Methylation 450 BeadChip in early-stage HCC patients 48. Han LY et al. discovered the value of DNA methylation at G-protein-coupled bile acid receptor Gpbar1 (TGR5) in ctDNA as a biomarker for HCC patients with chronic hepatitis B (CHB) 49. MT1M and MT1G methylation were detected in HCC patients´ serum with a significantly higher frequency compared to patients with CHB or the healthy control group 50. Occurring early in liver tumor progression with higher levels in HCC patients, INK4A promoter hyper-methylation also showed the potential as a biomarker 45.

VIM methylated gene repeatedly appeared as the research focus in several studies, manifesting its significance as a biomarker for ctDNA in HCC. Wen L et al. showed that four hypermethylated CpG islands (RGS10, ST8SIA6, RUNX2 and VIM) could be detected in blood and HCC tissue; simultaneously from dozens of high-performance markers of HCC by MCTA-Seq technique 43. In another study, a panel of genes with methylation of ctDNA was assessed in two different cohorts (France and Thailand), discovered that changes in VIM and FBLN1 methylation levels could represent effective ctDNA biomarkers 47. SEPT9 was also verified to be the circulating epigenetic biomarker in plasma for tumor diagnosis at an individual level 44.

In addition to sporadic individual genes, Xu RH et al. gathered several methylation points from two gene panels: one was developed as a diagnostic panel for HCC, which includes ten different methylation points: BMPR1A, PSD, ARHGAP25, KLF3, PLAC8, ATXN1, Chr 6:170, Chr 6:3, ATAD2. The other could predict prognosis, including SH3PXD2A, C11orf9, PPFIA1, Chr 17:78, SERPINB5, NOTCH3, GRHL2 and TMEM8B 51.

Discriminated with methylation, 5-methylcytosine (5mCs) are converted to 5hmCs by 5mC hydroxylase TET1, causing DNA demethylation alternation in mammalian cells. Recent research found out that 5hmC was decreased in HCC patients at a very early stage, which was mainly because of the decrease of 5mC associated with the hepatitis virus and activity of the TET enzyme 65. Therefore, 5hmC might be a biomarker of ctDNA with outstanding value for human cancer diagnosis and staging, including liver cancer, lung cancer, colorectal and gastric cancer, and pancreatic cancer 39,40.

**Figure 2:**
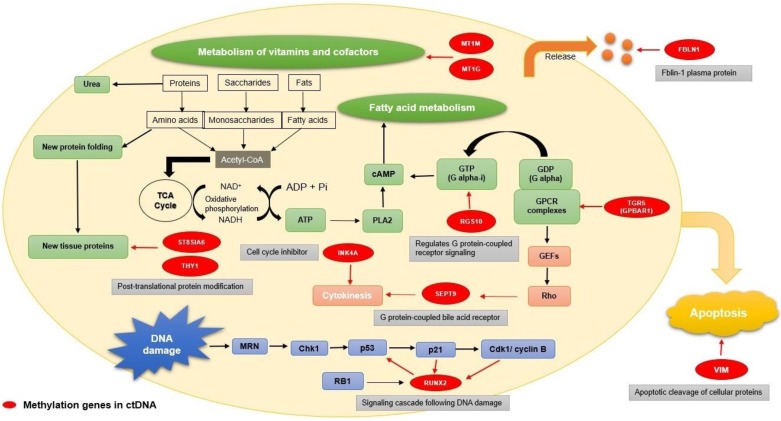
Functions of the genes with methylation. The functions of the genes with methylation (marked with red) mainly focus on several aspects: DNA damaged, metabolic regulation, apoptosis, G protein-coupled signal transmission, cell division, and some plasma protein release. RUNX2 has an influence on DNA damage(marked with blue); THY1, ST8SIA6, MT1M, MT1G, and RGS10 participate in the metabolism process (marked with green); VIM has a connection with apoptosis (marked with yellow); TGR5(GPBAR1) is a G-protein-coupled bile acid receptor for bile acid mediating; SEPT9 plays a critical role of cytokinesis and INK4A (CDKN2A) is a cell cycle inhibitor(marked with light red); FBLIN1 is related to plasma glycoprotein generation(marked with orange).

The methylated genes originally have different functions, including DNA damage, metabolic regulation, apoptosis, G protein-coupled signal transmission, and some plasma protein release (Fig. 2). RUNX2 is a critical factor for osteoblast differentiation, interacting with the p53 tumor suppressor gene for DNA damage signaling cascade 66. THY1 and ST8SIA6 are both involved in post-translational protein modification. As a glycophosphatidylinositol (GPI) anchored protein, GPI-THY1 can regulate signals impacting cell adhesion, differentiation and migration 67. ST8SIA6 is one of the sialyltransferases acting on N-linked glycosylation for post-translational protein modification 68. MT1M and MT1G, members of metallothioneins (MTs), play vital roles in metal homeostasis, metal detoxification, and metabolism of vitamins and cofactors 69. RGS10 is a GTPase-activating protein. It is a specific type of Gα subunits and can attenuate the signaling pathway for heterotrimeric G proteins. As a specific type of Gα subunits, RGS10 affects the production of cAMP, further indirectly affecting fatty acid metabolism 70. During apoptosis, vimentin (VIM) is cleaved by several caspases, which can produce pro-apoptotic amino-terminal production to amplify the cell death signal 71. Abundantly expressed in cell membranes of gallbladder epithelium, TGR5 (GPBAR1) is a G-protein-coupled bile acid receptor for bile acid mediating 72. Among the septin family, which is as GTP-binding proteins for cell division, SEPT9 is a key factor for cytokinesis and has linked alterations to cancer development 73. INK4A (CDKN2A), which belongs to the family of cyclin-dependent kinase inhibitors, leads to cell cycle arrest in the G1 phase by causing the inhibition of cyclin D-dependent kinases 74. Fibulin-1(FBLN1) belongs to an enormous family of plasma glycoprotein and will be significantly increased in acute liver injury 75.

#### CtDNA combines with protein markers

Recently, there was an interesting study by Cohen JD et al. for a new liquid biopsy method called “CancerSEEK” for tumor early detection, which combined mutations in ctDNA and circulating proteins for eight common cancer types (ovary, liver, stomach, pancreas, esophagus, colorectum, lung and breast). In 1005 cancer patients the test was positive in a median of 70% (ranging from 69 to 98%), and specificity was more than 99% for the different tumor entities, compared with 812 healthy controls. The sensitivity of liver cancer was nearly 98%. It reaches almost 100% in early tumor detection (for stage I HCC patients). Limitations of this study were the small size of a cohort including only 44 patients with liver cancer (39 hepatocellular carcinoma and 5 cholangiocarcinomas). This study showed how effective ctDNA could be applied for liver cancer detection 76.

### Clinical application of ctDNA detection in HCC patients in liquid biopsies

Future application of liquid biopsy represents a critical direction with broad clinical prospects. CtDNA has many advantages: non-invasive, multiple time points monitoring, characterization of cancer, identification of mechanisms of resistance. Compared with standard tumor biopsy, blood samples are easy to obtain multiple times in a non-invasive method. Thus, for patients who do not need or cannot undergo surgery, the non-invasive ctDNA analysis provides an option of molecular information. It allows a practical way of monitoring tumor change in patients serially over time without physical injury.

At present, diagnosis at late-stage and tumor recurrence remains to be a severe challenge in HCC treatment, because of due limited techniques for clinical use. A tumor is always caused by a gradual increase of genetic aberrations that manage cell proliferation and apoptotic. Thus ctDNA detection using genetic changes as a detection indicator has the huge potential to detect tumor cells at early stages. In the research of Xu RH et al, ctDNA methylation markers showed different scores between early-stage disease (I, II) and advanced-stage disease (III, IV) 77, reflecting the potential of ctDNA to be an early diagnostic tool for HCC indirectly. It is also in multiple tumors at early stages as well, including colorectal, ovarian, lung, and breast cancer 78. Moreover, tumor cell residual is one of the important factors for postoperative tumor relapse. Owing to the short half-life of ctDNA, any changes in tumor-derived ctDNA are able to provide clear evidence of real-time development of carcinoma, and this could help to detect postoperative tumor residuals and guide following treatment. As ctDNA correlates with tumor burden, it could be a tool for recurrence monitoring for HCC.

CtDNA is an emerging technique with immense potential for different clinical applications in HCC such as early tumor detection, therapy evaluation or monitoring of metastasis. Therefore, ctDNA may reflect tumor heterogeneity and subclone mutation during disease progression. Gene alteration could be made mutation or methylation panel to assess the prognosis in gene profiling 51. Precision oncology is defined as seeking targeted molecular profiling and therapy on carcinoma to improve the patient's prognosis. It aims to deliver the appropriate personalized cancer treatment to each individual in terms of techniques, time and dose. The merits of ctDNA which we have discussed above in this article show the potential of its promising applications in precision oncology 79.

### Future prospects

TACE is another first-line treatment for HCC patients, aiming to induce tumor necrosis 80. Simultaneously, tumor necrosis is also one of the mechanisms of ctDNA release from the cell. Thus, in the future, the variation of ctDNA may be compelling data for whether to choose TACE as a supplementary treatment after surgery and the timing of TACE treatment. Simultaneously, profiling the molecular changes in ctDNA/cfDNA may be able to guide targeted therapy. Sorafenib, a multikinase inhibitor for anti-angiogenesis and anti-proliferation, can prolong nearly 3 months of median survival time for advanced HCC patients. HCC patients receiving sorafenib treatment show the amplification of VEGFA copy number 28. CtDNA may help to monitor the efficacy of treatment and time to detect drug resistance for HCC patients 81.

CtDNA has also the potential for distinguishing tumor subtypes. This could be vital to guide diagnosis and therapy. Right now there are no relevant studies for HCC ctDNA differentiation of subtypes. However, the ctDNA genotype has been proven to classify tumor subtypes in diffuse large B cell lymphoma 82. In lung cancer, ctDNA may detect two different subtypes in the EGFR mutant gene 83. Thus it is necessary and urgent to explore the potential of HCC-ctDNA regarding these aspects.

Simultaneously, the exploration of genetic aberration in HCC ctDNA mainly depends on known genetic changes in tumor tissues at present. It has certain limitations: detection rates of mutated genes in different populations in ctDNA did not match detection rates in tumor tissues. The increasing development of artificial intelligence (AI), in medical imaging for cancer patients (e.g. lung, brain, breast, and prostate cancer 84) could be combined with database of HCC tumor DNA, ctDNA and HCC marker proteins with AI could lead to exploring new biomarkers, establishing precision oncology detection, diagnosis, therapy and monitoring for patients.

### Conclusions

In this review, we summarized the studies on biomarkers for the detection of ctDNA from a normal cfDNA background in HCC patients in past the 5 years: CNVs, gene integrity, gene mutations, DNA methylation, and cfDNA combined with protein markers. In liver cancer research, the number of studies on methylation ranks the highest, followed by studies about gene mutation. Rather than being limited to ctDNA quantity and size, exploring ctDNA from the perspective of alterations in DNA genetic and epigenetic information has become a hot topic. These biomarkers offer new opportunities to improve the sensitivity and specificity of ctDNA analysis and contribute to achieving ctDNA technology for clinical applications. However, the challenge for current studies of ctDNA in HCC is the limitation of clearly defined useful genetic targets and biomarkers, which can help to distinguish circulating tumor DNA from normal cell-free DNA.

CtDNA detection has still several limitations: results of ctDNA analysis dependent on different extraction sources, methods, sample size, blood collection time and conditions difference between primary and recurrent tumors 85. Standardization of all these factors in different studies, as well as the procedure for blood collection and storage, will be keys for guaranteeing consistency of results. Despite the high incidence of liver cancer and promising results, the number of patients included in ctDNA clinical trials is still limited. Therefore, more researches are expected to take further exploration of more accurate biomarkers of ctDNA or different combinations of ctDNA with other effective markers.

## Supplementary Material

Supplementary appendix.

## Figures and Tables

**Table 1:** Different biomarkers of ctDNA for HCC

Patients	Controls	Ethnicity	Sample	Sample vol.	Biomarkers	Biomarker Type	Positive Rate (%)	Supplement information	Ref.
24	62 (HBV)	China	Plasma	1ml	Concentration	CfDNA level	-	-	25
31	8 (CLD)	China	Plasma	-	CNVs & SNVs	CNVs	-	-	26
34	0	China	Plasma	-	CNV	CNVs	-	CNVs correlating to tumor burden	27
151	14 (HV)	Korea	Plasma	1.5ml	VEGFA & CNV	Amplication & CNVs	-	-	28
90	67 (CLD) 36 (LC) 32 (HV)	China	Plasma	3-4.8 ml	CfDNA size	Integrity	-	-	29
53	16 (OLT)	China	Plasma	1ml	Plasma DNA integrity	Integrity	-	ALU as primer	30
-	-	China	Plasma	4ml	Preferred plasma DNA end coordinates	Integrity	-	-	31
27	0	Asia, Europe, USA	Plasma	-	RAS (KRAS & NRAS);	Mutation	44.4	Evaluated for RAS mutational status by BEAMing firstly	32
					TERT	Mutation	63.0		
					TP53	Mutation	48.1		
					CTNNB1	Mutation	37.0		
66	35 (LC) 41 (CLD)	Italy	Plasma	200ul	TERT	Mutation	-	-	33
7	0	Europe	Plasma	3-6ml	TERT TP53 CTNNB1 TSC1 RB1 APOB et al.	Mutation	86	The large tumor was>5 cm or metastatic HCC	34
23	0						9	The small (largest tumor<5 cm), nonmetastatic HCC	
66	0	China	Plasma	5ml	TP53 CTNNTB1 AXIN1 ARID1A	Mutation	60 15.7 14.3 14.3	-	35
51	10 (LC)	UK & Italy	Plasma	-	ARID1A CTNNB1 TP53	Mutation	11.7 7.8 7.8	-	36
29	0	China	Plasma	1.5-1.8ml	TP53 ATM ALK NPM1 CSF1R KIT ERBB4 SMAD4 FBXW7 PTEN	Mutation	50 39 36 36 36 32 32 29 29	-	37
33	0	China	Plasma	5-6ml	TP53 CTNNB1 AXIN1 JAK1 EPS15 CACNA2D4	Mutation	52-84	-	38
206	0	USA	Plasma	5-6ml	TP53 EGFR MET ARID1A MYC NF1 BRAF ERBB2	Mutation & Amplification	0.49 (range 0.06 - 55.03%)	median mutant allele frequency (% cfDNA)	24
26	0	USA	Plasma	-	5hmC	Methylation	-	-	39
25	90 (HV)	China	Plasma	2ml	5hmC	Methylation	44	-	40
1204	392 (CLD or LC) 958 (HV and BT)	China	Plasma	3-6ml	5hmC	Methylation	-	validation set: area under curve (AUC)=88.4%	41
29	32 (HV) 8 (HBV)	USA	Plasma	5ml	multiple CpG sites	Methylation	94.8	-	42
36	38 (HV;LC; CLD)	China	Serum	2ml	RGS10 ST8SIA6 RUNX2 VIM	Methylation	94	-	43
51	186 (LC)	France	Plasma	3.5ml	SEPT9	Methylation	94.1	Initial Study	44
47	103 (LC)	Germany					85.1	Replication Study	
66	43 (CLD)	United States	Serum	1-2ml	INK4A	Methylation	65	-	45
8	8 (HV)	France	Plasma	1ml	VIM	Methylation	2.3	-	46
					FBLN1		-		
32	38 (HV)	France	Plasma	1ml	VIM	Methylation	1.48	Odds ratios	47
					FBLN1		0.89		
22	16 (CLD) 28 (HV)	Thailand	Plasma	1ml	VIM	Methylation	2.18		
					FBLN1		0.75		
31	27 (HV) 31 (HBV)	China	Serum	-	DBX2	Methylation	88	-	48
					THY1		85		
160	88 (CLD) 45 (HV)	China	Serum	400ul	TGR5	Methylation	48	-	49
121	37 (CLD) 31 (HV)	China	Serum	400ul	MT1M	Methylation	84	-	50
					MT1G	Methylation	70		
715	560 (HV)	China	Plasma	1.5ml	BMPR1A, PSD, ARHGAP25, KLF3, PLAC8, ATXN1, Chr 8:20, Chr 6:170, Chr 6:3, ATAD2	Methylation	85.7	Diagnostic Panel	51
1049	-	China	Plasma	1.5ml	SH3PXD2A, C11orf9, PPFIA1, SERPINB5, Chr 17:78, NOTCH3, GRHL2, TMEM8B	Methylation	-	Prognostic prediction Panel	

Different kinds of biomarkers have been used to detect ctDNA from normal cfDNA, including cfDNA level, DNA copy number, gene integrity, gene mutations, and DNA methylation alterations. In the past 5 years of ctDNA biomarker research, DNA methylation has become a research hotspot, followed by genetic mutation.

## References

[1] LLOVET JM, BRU C, BRUIX J (1999). Prognosis of hepatocellular carcinoma: The bclc staging classification.

[2] Lang H, Sotiropoulos GC, Brokalaki EI, Schmitz KJ, Bertona C, Meyer G (2007). Survival and recurrence rates after resection for hepatocellular carcinoma in noncirrhotic livers. Journal of the american college of surgeons.

[3] Trevisani F, D'Intino PE, Morselli-Labate AM, Mazzella G, Accogli E, Caraceni P (2001). Serum a-fetoprotein for diagnosis of hepatocellular carcinoma in patients with chronic liver disease: influence of HBsAg and anti-HCV status. Journal of Hepatology.

[4] Okajima W, Komatsu S, Ichikawa D, Miyamae M, Ohashi T, Imamura T (2017). Liquid biopsy in patients with hepatocellular carcinoma: Circulating tumor cells and cell-free nucleic acids. World J Gastroenterol.

[5] Villanueva A (2019). Hepatocellular carcinoma. The New England Journal of Medicine.

[6] Li J, Han X, Yu XN, Xu ZZ, Yang GS, Liu BQ (2018). Clinical applications of liquid biopsy as prognostic and predictive biomarkers in hepatocellular carcinoma: Circulating tumor cells and circulating tumor DNA. Journal of Experimental & Clinical Cancer Research.

[7] Felden Jv, Craig AJ, Villanueva A (2018). Role of circulating tumor DNA to help decision-making in hepatocellular carcinoma. Oncoscience.

[8] Mandel P, Metais P (1948). Les acides nucléiques du plasma sanguin chez l'homme.

[9] Fan HC, Blumenfeld YJ, Chitkara U, Hudgins L, Quake SR (2010). Analysis of the size distributions of fetal and maternal cell-free DNA by paired-end sequencing. Clinical Chemistry.

[10] Leon SA, Shapiro B, Sklaroff DM, Yaros MJ (1977). Free DNA in the serum of cancer patients and the effect of therapy. Cancer research.

[11] Corcoran RB, Chabner BA (2018). Application of cell-free DNA analysis to cancer treatment. The New England Journal of Medicine.

[12] Zinkova A, Brynychova I, Svacina A, Jirkovska M, Korabecna M (2017). Cell-free DNA from human plasma and serum differs in content of telomeric sequences and its ability to promote immune response. Scientific Reports.

[13] Martignano F (2019). Cell-Free DNA: An Overview of Sample Types and Isolation Procedures. Methods Mol Biol.

[14] van Dessel LF, Beije N, Helmijr JC, Vitale SR, Kraan J, Look MP (2017). Application of circulating tumor DNA in prospective clinical oncology trials - standardization of preanalytical conditions. Mol Oncol.

[15] Sherwood JL, Corcoran C, Brown H, Sharpe AD, Musilova M, Kohlmann A (2016). Optimised pre-analytical methods improve kras mutation detection in circulating tumour DNA (ctDNA) from patients with non-small cell lung cancer (nsclc). PLoS One.

[16] Li SP, Bartlett B, Popoli M, Adleff V, Tucker L, Steinberg R (2017). The effect of preservative and temperature on the analysis of circulating tumor DNA. Clinical Cancer Research.

[17] Sorber L, Zwaenepoel K, Deschoolmeester V, Roeyen G, Lardon F, Rolfo C (2017). A comparison of cell-free DNA isolation kits: Isolation and quantification of cell-free DNA in plasma. The Journal of Molecular Diagnostics.

[18] Elazezy M, Joosse SA (2018). Techniques of using circulating tumor DNA as a liquid biopsy component in cancer management. Computational and Structural Biotechnology Journal.

[19] Garcia-Foncillas J, Alba E, Aranda E, Diaz-Rubio E, Lopez-Lopez R, Tabernero J (2017). Incorporating BEAMing technology as a liquid biopsy into clinical practice for the management of colorectal cancer patients: an expert taskforce review. Annals of Oncology.

[20] Shendure J, Ji H (2008). Next-generation DNA sequencing. Nature Biotechnology.

[21] Hindson BJ, Ness KD, Masquelier DA, Belgrader P, Heredia NJ, Makarewicz AJ (2011). High-throughput droplet digital PCR system for absolute quantitation of DNA copy number. Analytical Chemistry.

[22] Pasternack H, Fassunke J, Plum PS, Chon SH, Hescheler DA, Gassa A (2018). Somatic alterations in circulating cell-free DNA of oesophageal carcinoma patients during primary staging are indicative for post-surgical tumour recurrence. Sci Rep.

[23] Newman AM, Bratman SV, To J, Wynne JF, Eclov NC, Modlin LA (2014). An ultrasensitive method for quantitating circulating tumor DNA with broad patient coverage. Nature Medicine.

[24] Kaseb AO, Sánchez NS, Sen S, Kelley RK, Tan B, Bocobo AG (2019). Molecular profiling of hepatocellular carcinoma using circulating cell-free DNA.

[25] Yan LL, Chen YH, Zhou JY, Zhao H, Zhang HH, Wang GQ (2018). Diagnostic value of circulating cell-free DNA levels for hepatocellular carcinoma. International Journal of Infectious Diseases.

[26] Xu H, Zhu X, Xu Z, Hu Y, Bo S, Xing T (2015). Non-invasive analysis of genomic copy number variation in patients with hepatocellular carcinoma by next generation DNA sequencing. Journal of Cancer.

[27] Cai Z, Chen G, Zeng Y, Dong X, Li Z, Huang Y (2019). Comprehensive liquid profiling of circulating tumor DNA and protein biomarkers in long-term follow-up patients with hepatocellular carcinoma. Clin Cancer Res.

[28] Oh CR, Kong SY, Im HS, Kim HJ, Kim MK, Yoon KA (2019). Genome-wide copy number alteration and VEGFA amplification of circulating cell-free DNA as a biomarker in advanced hepatocellular carcinoma patients treated with Sorafenib. BMC Cancer.

[29] Jiang P, Chan CWM, Chan KCA, Cheng SH, Wong J, Wong VW-S (2015). Lengthening and shortening of plasma DNA in hepatocellular carcinoma patients.

[30] Huang A, Zhang X, Zhou SL, Cao Y, Huang XW, Fan J (2016). Plasma Circulating Cell-free DNA Integrity as a Promising Biomarker for Diagnosis and Surveillance in Patients with Hepatocellular Carcinoma. Journal of Cancer.

[31] Jiang P, Sun K, Tong YK, Cheng SH, Cheng THT, Heung MMS (2018). Preferred end coordinates and somatic variants as signatures of circulating tumor DNA associated with hepatocellular carcinoma.

[32] Lim HY, Merle P, Weiss KH, Yau T, Ross P, Mazzaferro V (2018). Phase II Studies with Refametinib or Refametinib plus Sorafenib in Patients with RAS-Mutated Hepatocellular Carcinoma. Clinical Cancer Research.

[33] Piciocchi M, Cardin R, Vitale A, Vanin V, Giacomin A, Pozzan C (2013). Circulating free DNA in the progression of liver damage to hepatocellular carcinoma. Hepatology International.

[34] Ng CKY, Di Costanzo GG, Tosti N, Paradiso V, Coto-Llerena M, Roscigno G (2018). Genetic profiling using plasma-derived cell-free DNA in therapy-naive hepatocellular carcinoma patients: a pilot study. Annals of Oncology.

[35] Huang A, Zhao X, Yang XR, Li FQ, Zhou XL, Wu K (2017). Circumventing intratumoral heterogeneity to identify potential therapeutic targets in hepatocellular carcinoma. Journal of Hepatology.

[36] Howell J, Atkinson SR, Pinato DJ, Knapp S, Ward C, Minisini R (2019). Identification of mutations in circulating cell-free tumour DNA as a biomarker in hepatocellular carcinoma. European Journal of Cancer.

[37] He G, Chen Y, Zhu C, Zhou J, Xie X, Fei R (2019). Application of plasma circulating cell free DNA detection to the molecular diagnosis of hepatocellular carcinoma.

[38] Xiong Y, Xie CR, Zhang S, Chen J, Yin ZY (2019). Detection of a novel panel of somatic mutations in plasma cell-free DNA and its diagnostic value in hepatocellular carcinoma. Cancer Management and Research.

[39] Song CX, Yin SL, Ma L, Wheeler A, Chen Y, Zhang Y (2017). 5-hydroxymethylcytosine signatures in cell-free DNA provide information about tumor types and stages. Cell Research.

[40] Li W, Zhang X, Lu X, You L, Song Y, Luo Z (2017). 5-Hydroxymethylcytosine signatures in circulating cell-free DNA as diagnostic biomarkers for human cancers. Cell Research.

[41] Cai J, Chen L, Zhang Z, Zhang X, Lu X, Liu W (2019). Genome-wide mapping of 5-hydroxymethylcytosines in circulating cell-free DNA as a non-invasive approach for early detection of hepatocellular carcinoma. Gut.

[42] Li W, Li Q, Kang S, Same M, Zhou Y, Sun C (2018). Cancerdetector: Ultrasensitive and non-invasive cancer detection at the resolution of individual reads using cell-free DNA methylation sequencing data. Nucleic Acids Research.

[43] Wen L, Li J, Guo H, Liu X, Zheng S, Zhang D (2015). Genome-scale detection of hypermethylated CpG islands in circulating cell-free DNA of hepatocellular carcinoma patients. cell Research.

[44] Oussalah A, Rischer S, Bensenane M, Conroy G, Filhine-Tresarrieu P, Debard R (2018). Plasma mSEPT9: A novel circulating cell-free DNA-based epigenetic biomarker to diagnose hepatocellular carcinoma.

[45] Huang G, Krocker JD, Kirk JL, Merwat SN, Ju H, Soloway RD (2014). Evaluation of INK4A promoter methylation using pyrosequencing and circulating cell-free DNA from patients with hepatocellular carcinoma. Clinical Chemistry Labratory Medicine.

[46] Vaca-Paniagua F, Oliver J, Nogueira da Costa A, Merle P, McKay J, Herceg Z (2015). Targeted deep DNA methylation analysis of circulating cell-free DNA in plasma using massively parallel semiconductor sequencing. Epigenomics.

[47] Holmila R, Sklias A, Muller DC, Esposti DD, Guilloreau P, McKay J (2017). Targeted deep sequencing of plasma circulating cell-free DNA reveals Vimentin and Fibulin 1 as potential epigenetic biomarkers for hepatocellular carcinoma. PLoS One.

[48] Zhang PJ, Wen XY, Gu F, Deng XX, Li J, Dong J (2013). Methylation profiling of serum DNA from hepatocellular carcinoma patients using an infinium human methylation 450 beadchip. Hepatology International.

[49] Han LY, Fan YC, Mu NN, Gao S, Li F, Ji XF (2014). Aberrant DNA methylation of g-protein-coupled bile acid receptor gpbar1 (TGR5) is a potential biomarker for hepatitis b virus associated hepatocellular carcinoma. International Journal of Medical Sciences.

[50] Ji XF, Fan YC, Gao S, Yang Y, Zhang JJ, Wang K (2014). MT1M and MT1G promoter methylation as biomarkers for hepatocellular carcinoma. World J Gastroenterology.

[51] Xu RH, Wei W, Krawczyk M, Wang W, Luo H, Flagg K (2017). Circulating tumour DNA methylation markers for diagnosis and prognosis for diagnosisandprognosis of hepatocellular carcinoma.

[52] Cheng J, Holland-Letz T, Wallwiener M, Surowy H, Cuk K, Schott S (2018). Circulating free DNA integrity and concentration as independent prognostic markers in metastatic breast cancer. Breast Cancer Res Treat.

[53] Cohen JD, Javed AA, Thoburn C, Wong F, Tie J, Gibbs P (2017). Combined circulating tumor DNA and protein biomarker-based liquid biopsy for the earlier detection of pancreatic cancers. Proc Natl Acad Sci U S A.

[54] Lee SE, Chang SH, Kim WY, Hwang TS, Kim WY, Han HS (2016). Frequent somatic TERT promoter mutations and CTNNB1 mutations in hepatocellular carcinoma.

[55] Network (2017). CGAR. Comprehensive and Integrative Genomic Characterization of Hepatocellular Carcinoma. Cell.

[56] Whittaker S, Marais R, Zhu AX (2010). The role of signaling pathways in the development and treatment of hepatocellular carcinoma. Oncogene.

[57] Gollob JA, Wilhelm S, Carter C, Kelley SL (2006). Role of Raf kinase in cancer: therapeutic potential of targeting the Raf/MEK/ERK signal transduction pathway. Semin Oncol.

[58] Downward J (2003). Targeting RAS signalling pathways in cancer therapy. Nat Rev Cancer.

[59] Pinyol R, Tovar V, Llovet JM (2014). TERT promoter mutations: gatekeeper and driver of hepatocellular carcinoma. Journal of Hepatology.

[60] Hussain SP, Schwank J, Staib F, Wang XW, Harris CC (2007). TP53 mutations and hepatocellular carcinoma: insights into the etiology and pathogenesis of liver cancer. Oncogene.

[61] Zhan T, Rindtorff N, Boutros M (2017). Wnt signaling in cancer. Oncogene.

[62] Kimelman D, Xu W (2006). β-Catenin destruction complex: insights and questions from a structural perspective. Oncogene.

[63] Abitbol S, Dahmani R, Coulouarn C, Ragazzon B, Mlecnik B, Senni N (2018). AXIN deficiency in human and mouse hepatocytes induces hepatocellular carcinoma in the absence of beta-catenin activation. Journal of Hepatology.

[64] Sun X, Wang SC, Wei Y, Luo X, Jia Y, Li L (2017). Arid1a Has Context-Dependent Oncogenic and Tumor Suppressor Functions in Liver Cancer. Cancer Cell.

[65] Liu J, Jiang J, Mo J, Liu D, Cao D, Wang H (2019). Global DNA 5-Hydroxymethylcytosine and 5-Formylcytosine Contents Are Decreased in the Early Stage of Hepatocellular Carcinoma. Hepatology.

[66] Wysokinski D, Blasiak J, Pawlowska E (2015). Role of RUNX2 in Breast Carcinogenesis. International journal of molecular sciences.

[67] Bradley JE, Chan JM, Hagood JS (2013). Effect of the GPI anchor of human Thy-1 on antibody recognition and function. Lab Invest.

[68] Angata K, Fukuda M (2003). Polysialyltransferases: major players in polysialic acid synthesis on the neural cell adhesion molecule. Biochimie.

[69] Si M, Lang J (2018). The roles of metallothioneins in carcinogenesis.

[70] Hunt TW, Fields TA, Casey PJ, Peralta EG (1996). RGS10 is a selective activator of Gαi GTPase activity.

[71] Byun Y, Chen F, Chang R, Trivedi M, Green KJ, Cryns VL (2001). Caspase cleavage of vimentin disrupts intermediate filaments and promotes apoptosis.

[72] Pols TWH, Noriega LG, Nomura M, Auwerx J, Schoonjans K (2011). The bile acid membrane receptor TGR5: A valuable metabolic target. Digestive Diseases.

[73] Estey MP, Di Ciano-Oliveira C, Froese CD, Bejide MT, Trimble WS (2010). Distinct roles of septins in cytokinesis: SEPT9 mediates midbody abscission. The Journal of Cell Biology.

[74] Roussel MF (1999). The INK4 family of cell cycle inhibitors in cancer.

[75] Piscaglia F, Dudás J, Knittel T, Di Rocco P, Kobold D, Saile B (2009). Expression of ECM proteins fibulin-1 and -2 in acute and chronic liver disease and in cultured rat liver cells. Cell and Tissue Research.

[76] Cohen JD, Li L, Wang YX, Thoburn C, Afsari B, Danilova L (2018). Detection and localization of surgically resectable cancers with a multi-analyte blood test.

[77] Xu R-h, Wei W, Krawczyk M, Wang W, Luo H, Flagg K (2017). Circulating tumour DNA methylation markers for diagnosis and prognosis of hepatocellular carcinoma. Nature Materials.

[78] Phallen J, Sausen M, Adleff V, Leal A, Hruban C, White J (2017). Direct detection of early-stage cancers using circulating tumor DNA.

[79] Heitzer E, Haque IS, Roberts CES, Speicher MR (2019). Current and future perspectives of liquid biopsies in genomics-driven oncology. Nature Reviews Genetics.

[80] Raoul JL, Forner A, Bolondi L, Cheung TT, Kloeckner R, de Baere T (2019). Updated use of TACE for hepatocellular carcinoma treatment: How and when to use it based on clinical evidence. Cancer Treatment Reviews.

[81] Llovet JM, Ricci S, Mazzaferro V, Hilgard P, Gane E, Blanc J-F (2008). Sorafenib in advanced hepatocellular carcinoma.

[82] Scherer F, Kurtz DM, Newman AM, Stehr H, Craig AF, Esfahani MS (2016). Distinct biological subtypes and patterns of genome evolution in lymphoma revealed by circulating tumor DNA. Science Translational Medicine.

[83] Lyu M, Zhou J, Ning K, Ying B (2019). The diagnostic value of circulating tumor cells and ctDNA for gene mutations in lung cancer. OncoTargets and Therapy.

[84] Bi WL, Hosny A, Schabath MB, Giger ML, Birkbak NJ, Mehrtash A (2019). Artificial intelligence in cancer imaging: Clinical challenges and applications. CA Cancer J Clin.

[85] Yin CQ, Yuan CH, Qu Z, Guan Q, Chen H, Wang FB (2016). Liquid biopsy of hepatocellular carcinoma: Circulating tumor-derived biomarkers. Disease Markers.

