# Supplementary material for: Circulating tumor DNA as an emerging liquid biopsy biomarker for early diagnosis and therapeutic monitoring in hepatocellular carcinoma
Source: Int J Biol Sci. 2020 Mar 5;16(9):1551–62. doi: 10.7150/ijbs.44024 (PMC7097921; doi:10.7150/ijbs.44024)
Supplement: Supplementary file 1 — Supplementary appendix. [file ijbsv16p1551s1.pdf]

Appendix A

Abbreviations	Full name
ARID1A	AT-rich interactive domain-containing protein 1A
APOB	Apolipoprotein B
ARHGAP25	Rho GTPase activating protein 25
ATXN1	Ataxin 1
ATAD2	ATPase Family, AAA Domain Containing 2
AI	Artificial intelligence
BMPR1A	Bone morphogenetic protein receptor, type IA
BT	Benign tumor
CCND1	Cyclin D1
CLD	Chronic liver disease
CfDNA	Cell-free DNA
CtDNA	Circulating tumor DNA
CTCs	Circulating tumor cells
CTNNB1	Catenin beta 1
CNVs	Copy number variations
CDKN2A (INK4A)	Cyclin Dependent Kinase Inhibitor 2A
CHB	chronic hepatitis B
dd PCR	Digital droplet PCR
DBX2	Developing brain homeobox protein 2
FBLN1	Fibulin-1
GRHL2	Grainy head Like Transcription Factor 2
GPBAR1 (TGR5)	G Protein-Coupled Bile Acid Receptor 1
5hmC	5-Hydroxymethylcytosine
HCC	Hepatocellular carcinoma
HBV	Hepatitis B virus
HV	Healthy Volunteers
KRAS	KRAS Proto-Oncogene, GTPase
KLF3	Krüppel-like factor 3
LC	Liver cirrhosis
MAP	Mitogen-activated protein
MT1M	Metallothionein-1M
MT1G	Metallothionein-1G
MYRF (C11orf9)	Myelin Regulatory Factor
NGS	Next-generation sequencing
NRAS	NRAS Proto-Oncogene, GTPase
NOTCH3	Neurogenic Locus Notch Homolog Protein 3
OLT	Other malignant liver tumors
PCR	polymerase-chain-reaction
PSD	Pleckstrin And Sec7 Domain Containing
PPFIA1	PTPRF Interacting Protein Alpha 1
PLAC8	Placenta Specific 8
RAS	RAS GTPase
RB1	Retinoblastoma protein 1
RGS10	Regulator of G-protein signaling 10

RUNX2	Runt-related transcription factor 2
SWI/SNF	Switch/Sucrose Non-Fermentable
ST8SIA6	ST8 alpha-N-acetyl-neuraminide alpha-2,8-sialyltransferase 6
SEPT9	Septin-9
SH3PXD2A	SH3 And PX Domains 2A
SERPINB5	Serpin Family B Member 5
SIRT	Selective internal radiation therapy
TERT	Telomerase reverse transcriptase
TP53	Tumor protein p53
TSC1	Tuberous sclerosis 1
THY1	Thy-1 Cell Surface Antigen
TMEM8B	Transmembrane Protein 8B
TACE	Transarterial chemoembolization
VIM	Vimentin
